# Optimizing Peripheral Nerve Block Placement in Hip Surgery: A Cadaveric Study Mapping the Posterior Cutaneous Innervation

**DOI:** 10.1002/ca.24262

**Published:** 2025-02-14

**Authors:** Ziki Gurney, Kenneth Saw Kai Wei, Leia Boote, Daniel Gareth Stolady, Benjamin Fox, Alan R. Norrish

**Affiliations:** ^1^ School of Medicine Queen's Medical Centre University of Nottingham Nottingham UK; ^2^ Queen's Medical Centre, School of Life Sciences University of Nottingham Nottingham UK; ^3^ The Queen Elizabeth Hospital King's Lynn NHS Foundation Trust King's Lynn Norfolk UK

**Keywords:** cadaveric dissection, hip surgery, iliohypogastric nerve, ilioinguinal nerve, lateral approach, peripheral nerve block, posterior approach, posterolateral hip, subcostal nerve, ultrasound

## Abstract

Optimizing analgesia after hip surgery enables more rapid recovery. However, peripheral nerve blocks (PNBs) often fail to provide adequate pain relief in the posterolateral hip as they typically target the lateral cutaneous nerve of the thigh (LCNT). This study aimed to map the nerves innervating the posterolateral hip through analysis of anatomy textbooks (*n* = 5) and cadaveric dissections (*n* = 13). The subcostal (SCN), iliohypogastric (IHN), and ilioinguinal (IIN) nerves were identified as key contributors to innervating the posterolateral hip. The optimal site for ultrasound‐guided PNBs to target these three nerves was identified at the “75/25” landmark: 75% horizontally along the 12th rib and 25% vertically down to the iliac crest. Ultrasound‐guided dye injections in cadavers (*n* = 6) showed that while the “75/25” landmark effectively stained the SCN (6/6) and IHN (4/6), it inconsistently stained the IIN (2/6). A second injection in the posterolateral hip stained branches of the IHN (4/6) and IIN (4/6) but not the SCN (1/6), suggesting the IHN and IIN are the dominant nerves in the posterolateral hip. These findings recommend a more distal injection at the “100/75” landmark to consistently block the IHN and IIN, thereby optimizing postoperative analgesia after hip surgery.

## 1 Introduction

Peripheral nerve blocks (PNBs) involve injecting local anesthetic around nerves, typically identified using ultrasound (Guay and Kopp 2020), providing postoperative analgesia after the effects of general or regional anesthesia wear off (Schwenk and Mariano 2018). A single PNB injection can produce analgesic effects for up to 24 h, and its effects can be prolonged with continuous administration through a catheter (Ilfeld 2011). Despite their effectiveness, additional analgesics, such as paracetamol or opioids, may be necessary (National Institute for Health and Clinical Excellence 2011). However, opioids often cause adverse effects, particularly in elderly patients, such as nausea, vomiting, drowsiness, constipation, itching, difficulty urinating, and respiratory depression. These result in longer hospital stays and increased healthcare costs (Oderda et al. 2013). PNBs reduce postoperative opioid use facilitating more rapid recovery and discharge (Vandebroek et al. 2014).

**FIGURE 1:**
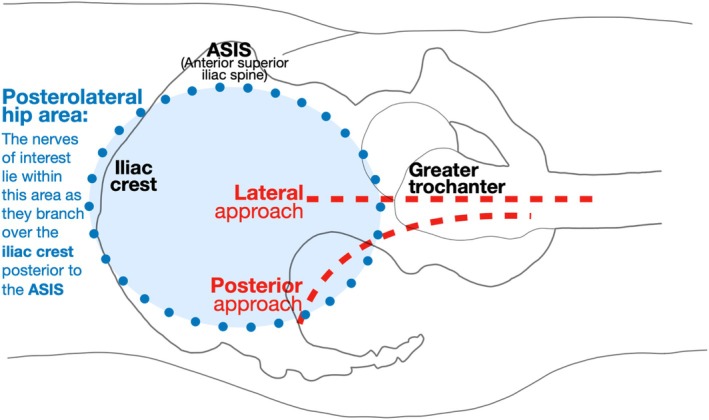
The posterior and lateral approaches to hip replacement surgery. Drawn on the lateral side of a right hip in supine position. Adapted from Glenister et Sharma (Glenister and Sharma 2022).

Hip surgeries are commonly approached via posterior or lateral incisions made within the posterolateral hip area (Li and Luo 2021; Glenister and Sharma 2022). This study focuses on the superficial cutaneous nerves in the posterolateral hip area, which branch over the iliac crest, posterior to the anterior superior iliac spine (ASIS) and toward the greater trochanter (Figure 1).

**FIGURE 2:**
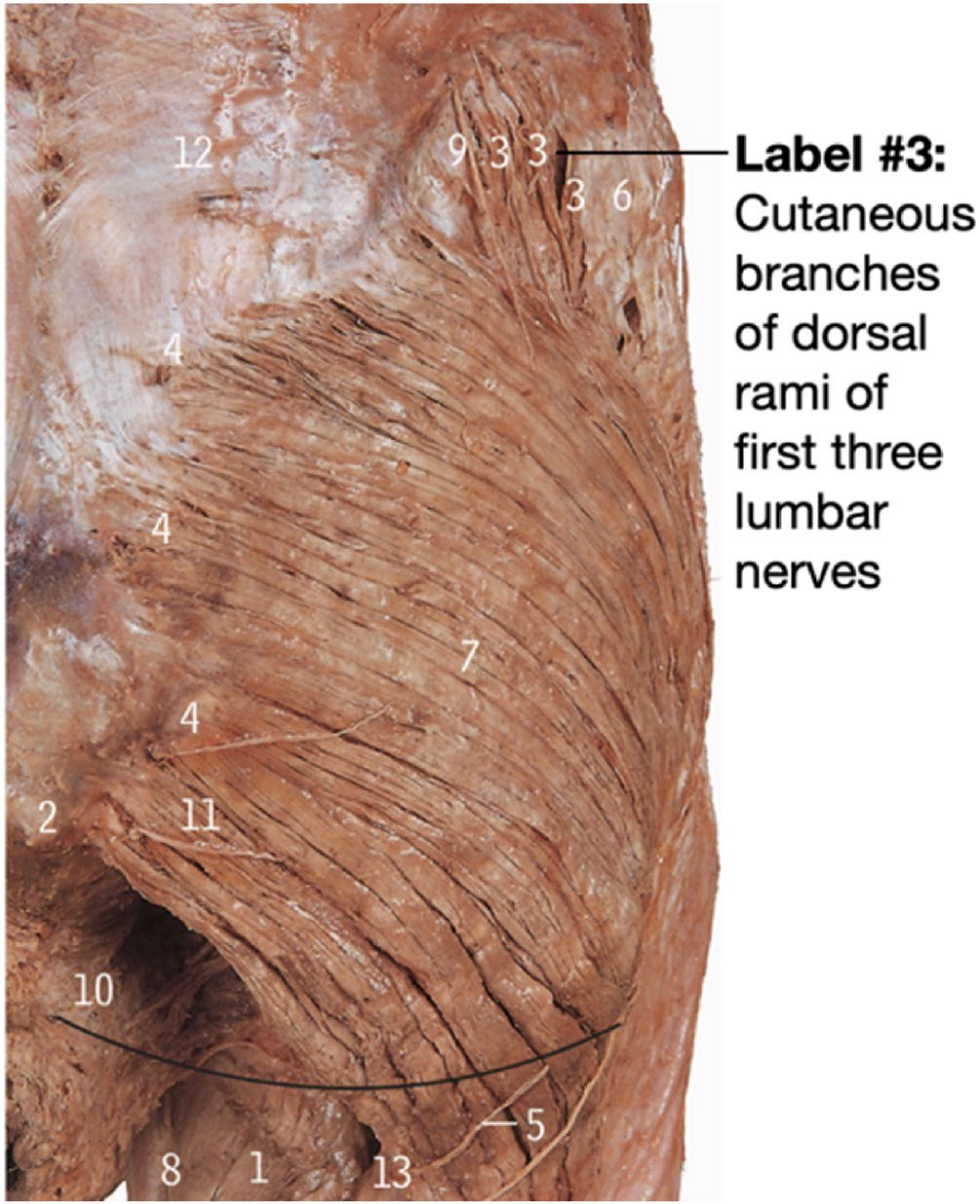
Superficial cutaneous nerves in the posterolateral hip area identified by Label 3. Adapted from Abraham's and McMinn's Clinical Atlas of Human Anatomy p.316 (Abrahams et al. 2019).

PNBs targeting the lateral cutaneous nerve of the thigh (LCNT), which is a purely sensory nerve, avoiding motor paralysis, are effective for the anterior aspect of the wound (Standring, Anand, and Tunstall 2021; Dalley and Agur 2024), but not the posterolateral edge of the wound (Davies et al. 2014; Nielsen et al. 2018). LCNT blocks often fail to reach the superficial cutaneous nerves in the posterolateral hip area (Figure 2, Label 3) (Nielsen et al. 2018; Abrahams et al. 2019), resulting in no significant pain relief compared to placebos (Thybo, Schmidt, and Hägi‐Pedersen 2015). Targeting these nerves in the posterolateral hip area may extend the area of anesthesia more superiorly and posteriorly than LCNT blocks alone.

**FIGURE 3:**
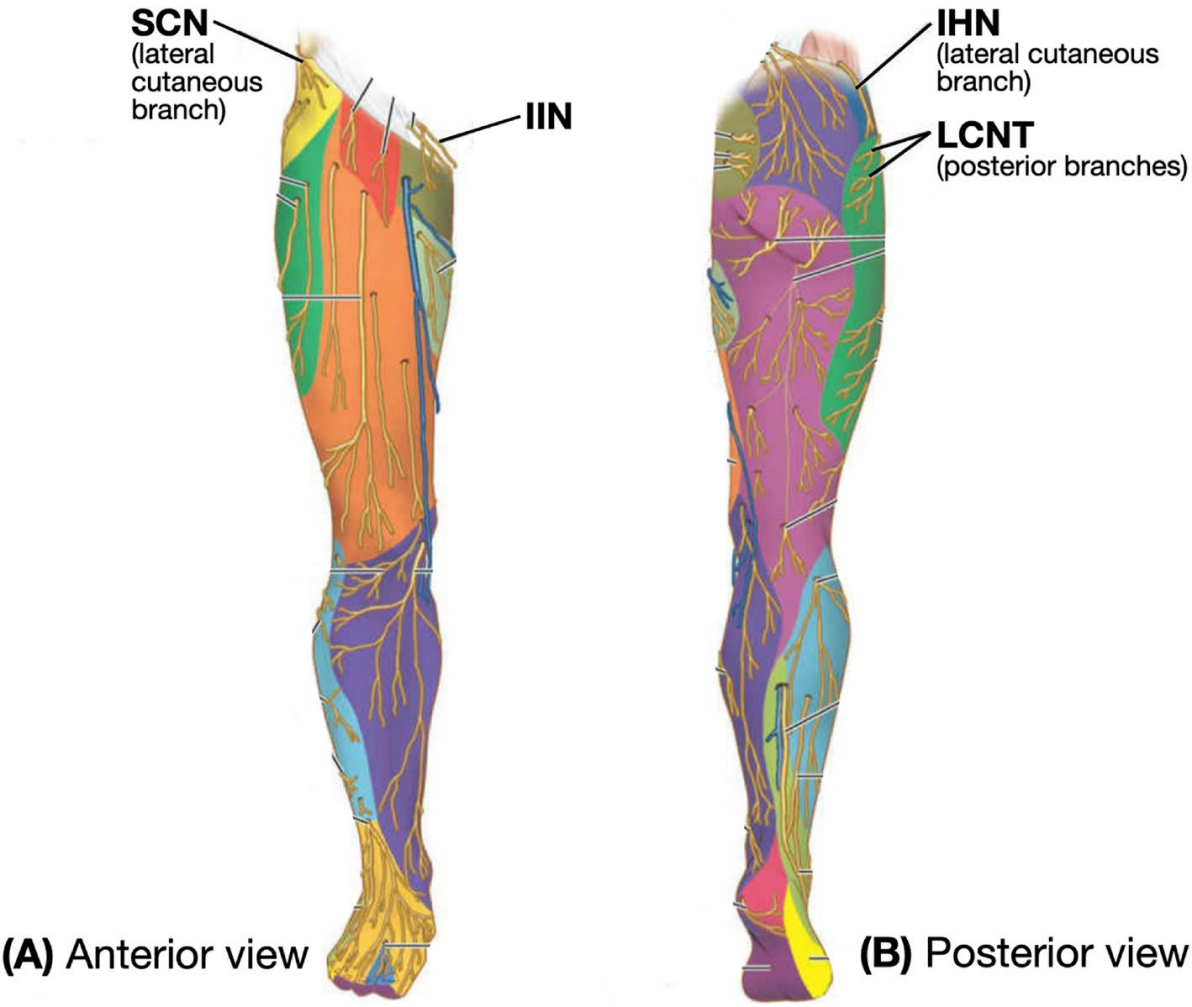
Superficial cutaneous nerves of lower limb. Nerves of interest are outlined in red. Adapted from Moore's Clinically Oriented Anatomy, p.702 (Dalley and Agur 2024).

Potential nerves innervating the posterolateral hip include the subcostal nerve (SCN), the iliohypogastric nerve (IHN), and the ilioinguinal nerve (IIN), highlighted in Figure 3 and detailed in Table 1 (Standring, Anand, and Tunstall 2021; Dalley and Agur 2024). While the lateral cutaneous branch of the IHN is often mapped in the posterolateral area, the SCN and IIN remain less understood (Glenister and Sharma 2022; Standring, Anand, and Tunstall 2021; Dalley and Agur 2024; Abrahams et al. 2019). Studies show the SCN to supply the skin below the iliac crest (Figure 3) (Maigne, Maigne, and Guerin‐Surville 1986), helping to extend the area of anesthesia beyond the LCNT block (Nielsen et al. 2019). Other findings suggest the IIN to innervate the iliac crest (Chin et al. 2012), with additional research identifying a lateral cutaneous branch of the IIN near the iliac crest (Apaydin and Bozkurt 2015; Drakonaki et al. 2022).

**TABLE 1:** Description of nerves of the three nerves of interest.

Nerve	Abbreviation	Origin from which spinal nerve root	Cutaneous innervation
Subcostal nerve	SCN	T12	Lateral cutaneous branch innervating hip area inferior to iliac crest and anterior to greater trochanter
Iliohypogastric nerve	IHN	L1 (occasionally T12)	Lateral cutaneous branch innervating superolateral buttock
Ilioinguinal nerve	IIN	L1 (occasionally T12)	Anteromedial thigh

*Source*: Adapted from Gray's Anatomy (Standring, Anand, and Tunstall 2021) and Moore's Clinically Oriented Anatomy p.703 (Dalley and Agur 2024).

The primary aim of this study was to map the course of the SCN, IHN, and IIN to identify potential injection sites for PNBs to provide analgesia in the posterolateral hip area. The secondary aim was to test the proposed injection sites to assess their efficacy in targeting the SCN, IHN, and IIN.

## 2 Materials and Methods

This descriptive cadaveric study used a mixed‐method approach, combining quantitative nerve mapping between two anatomical landmarks with qualitative descriptions of nerve courses and branching patterns.

### 2.1 Study Population

Cadavers were sourced from the Anatomy Suite in the School of Life Sciences at the University of Nottingham, with permission for further dissection. In total, 11 fresh–frozen and 2 embalmed hips were dissected, and 6 fresh–frozen hips were used for ultrasound‐guided dye injections. Cadaver characteristics are summarized in Table 2. Fresh–frozen cadavers, though less durable, are preferred for ultrasound‐guided dye injections as they offer a more realistic appearance, unaffected by the embalming process (Sawhney et al. 2017). In contrast, embalmed cadavers often show stiff soft tissue, increased vascular resistance and the presence of blood clots, making them less suitable for dye injections (Doomernik et al. 2016). Fresh–frozen cadavers were stored at −20°C and thawed before use, while embalmed cadavers were stored at −5°C and preserved in an embalming fluid comprising 71.8% ethanol, 10% phenol, 3.8% methanol, and 1.6% formaldehyde (Human Tissue Authority (HTA) 2015). No sample size calculation was performed as cadaver availability determined the sample size. Funding of £450 from The Queen Elizabeth Hospital King's Lynn NHS Foundation Trust Education Fund contributed to the cremation costs for the cadavers used in the study.

**TABLE 2:** Cadaver information.

Cadaver ID	Date of dissection	Sex	Height (cm)	Weight (kg)	Age at death (years)	Hip side dissected	Type
1	28/11/23	Female	156	80	95	Both	Fresh–frozen
2	08/12/23	Male	185	108	84	Right only	Fresh–frozen
3	20/02/24	Female	156	57	96	Both	Fresh–frozen
4	16/01/24	Male	175	67	98	Both	Embalmed
5	15/04/24	Female	180	Unknown	80	Both	Fresh–frozen
6	15/04/24	Female	165	56	76	Both	Fresh–frozen
7	15/04/24	Male	170	68	89	Both	Fresh–frozen

Inclusion criteria specified cadavers with an intact posterolateral area, age ≥ 18 years at death and consent for anatomical examination under the UK Human Tissue Act (2004). Exclusion criteria ruled out cadavers with hip pathologies or infectious diseases.

### 2.2 Mapping the Nerves

#### 2.2.1 Textbook Nerve Mapping

1. Anatomical textbooks consulted at QMC Greenfield Medical library and online through *NUSearch*.
2. Images representing the SCN, IHN, and IIN in posterior view with 12th rib and iliac crest exposed were selected.
3. Images uploaded to *ImageJ* for quantitative measurements.

#### 2.2.2 Cadaveric Nerve Mapping

The following steps outline the dissection for one hip and were repeated on the opposite side.

1. Preparation: cadaver placed pronely.
2. Dissection: SCN, IHN, and IIN identified between the 12th rib and iliac crest. The area that extends beyond these landmarks was dissected. Skin, fat, subcutaneous tissue, and fascia were removed to expose the underlying muscles and vasculature. Latissimus dorsi, thoracolumbar fascia, and erector spinae muscle were removed, leaving the quadratus lumborum muscle in situ. External and internal oblique muscle removed laterally, leaving transversus abdominis in situ.
3. Nerve identification: SCN, IHN, and IIN identified lateral to the quadratus lumborum and superficial to the abdominis.
4. Analysis: Scaled photographs from the posterior view were captured with a camera and uploaded to *ImageJ* for quantitative measurements.

#### 2.2.3 Ultrasound‐Guided Dye Injections of Fresh–Frozen Cadavers

The second part involved two ultrasound‐guided dye injections and subsequent dissection of fresh–frozen cadavers.

1. Dye preparation: 1 g of methylene blue powder dissolved in 400 mL water to create a 0.25% methylene blue solution. 5 mL of solution was drawn up using a syringe *(B Braun Omnifix)* and subsequently attached to a 50‐mm needle *(B Braun Stimuplex Ultra 360)*.
2. Cadaver preparation: Cadaver placed prone.
3. Ultrasound preparation: a linear probe is attached to the ultrasound machine *(GE LOGIQ V2)*. Ultrasound transmission gel *(Aquasonic 100) was* applied to the probe.
4. Injection 1: an ultrasound probe is placed at the ideal injection site determined by textbook and cadaveric mapping. Nerves were sonographically identified, then injected with 5 mL of 0.25% methylene blue. Experienced anesthetic ultrasonographers performed the injection to ensure quality assurance.
5. Injection 2: an ultrasound probe was placed at the level of the iliac crest posterior to the ASIS, and the injection site was refined depending on where nerves were sonographically visible. Similar to injection 1, 5 mL of 0.25% methylene blue was injected by experienced anesthetic ultrasonographers.
6. Dissection: the same method as “cadaveric nerve mapping.”
7. Analysis: SCN, IHN, and IIN and their branches were observed to determine dye staining from Injections 1 and 2.

### 2.3 Outcome Variables

**FIGURE 4:**
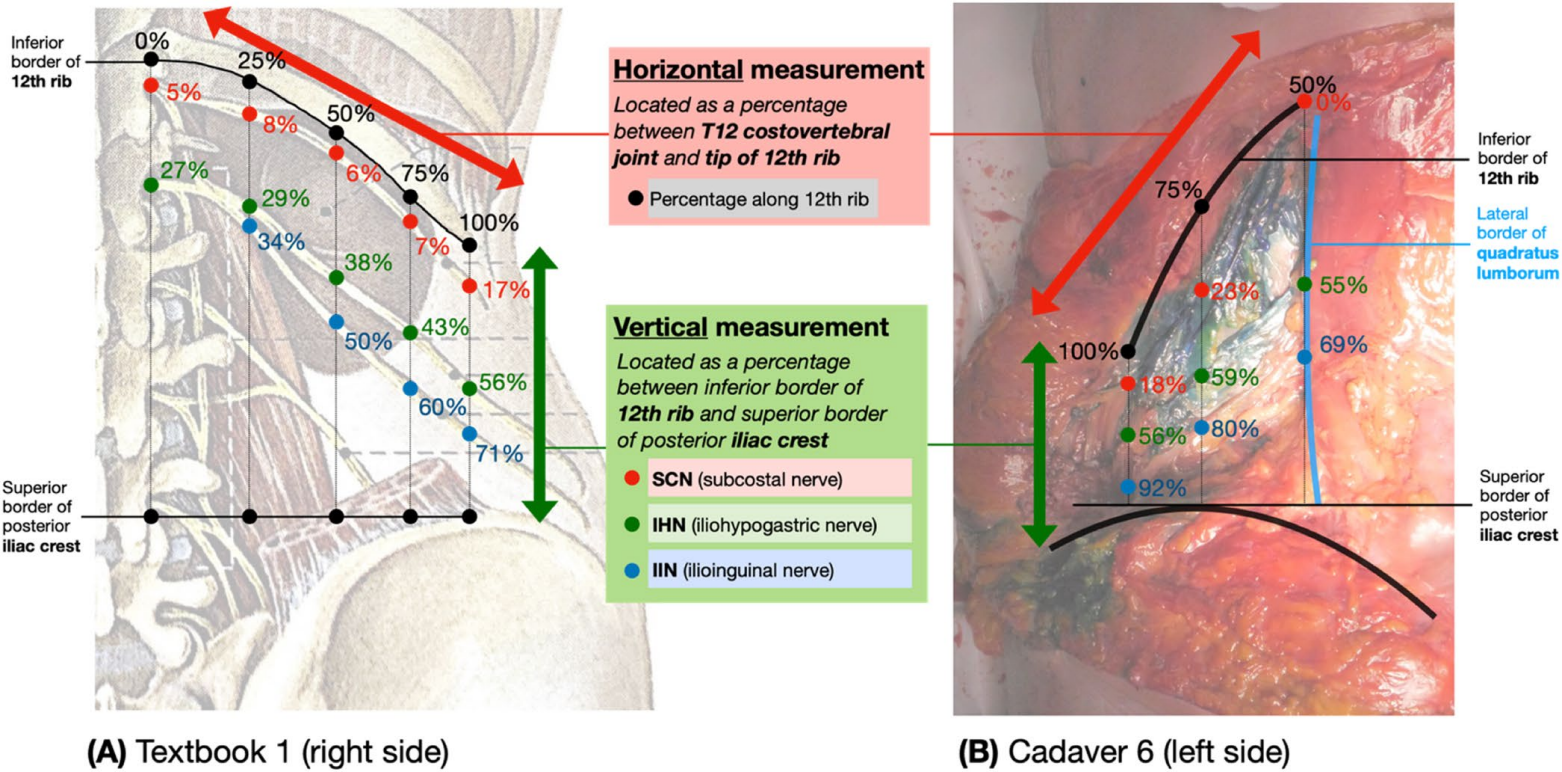
Example of vertical measurements taken at regular horizontal points along the 12th rib. (A) Adapted from Köpf‐Maier (Köpf‐Maier 2005). (B) Was an original photograph taken during dissection.

For textbook nerve mapping, vertical locations of the SCN, IHN, and IIN were measured as percentages between the 12th rib and iliac crest at five points along the 12th rib (0%, 25%, 50%, 75%, and 100%). Cadaveric nerve mapping focused on 50%, 75%, and 100% due to the quadratus lumborum muscle covering 0% and 25%. Percentages were calculated using *ImageJ* (Figure 4). Ultrasound‐guided dye injections were assessed using binary (yes/no) data on whether the nerves were stained based on direct observation.

### 2.4 Statistical Analysis

Measurements were made on *ImageJ* (NIH, USA), collected on Excel (Microsoft, USA), and analyzed using SPSS v29 (IBM, USA). To locate the nerves, mean and standard deviations (SDs) were calculated, with outliers identified using the interquartile range. Normality was assessed with the Shapiro–Wilk test. Two‐tailed unpaired *t*‐tests or Mann–Whitney *U* tests were used depending on normality, with significance set at *p* < 0.05.

## 3 Results

### 3.1 Textbook Nerve Mapping

**FIGURE 5:**
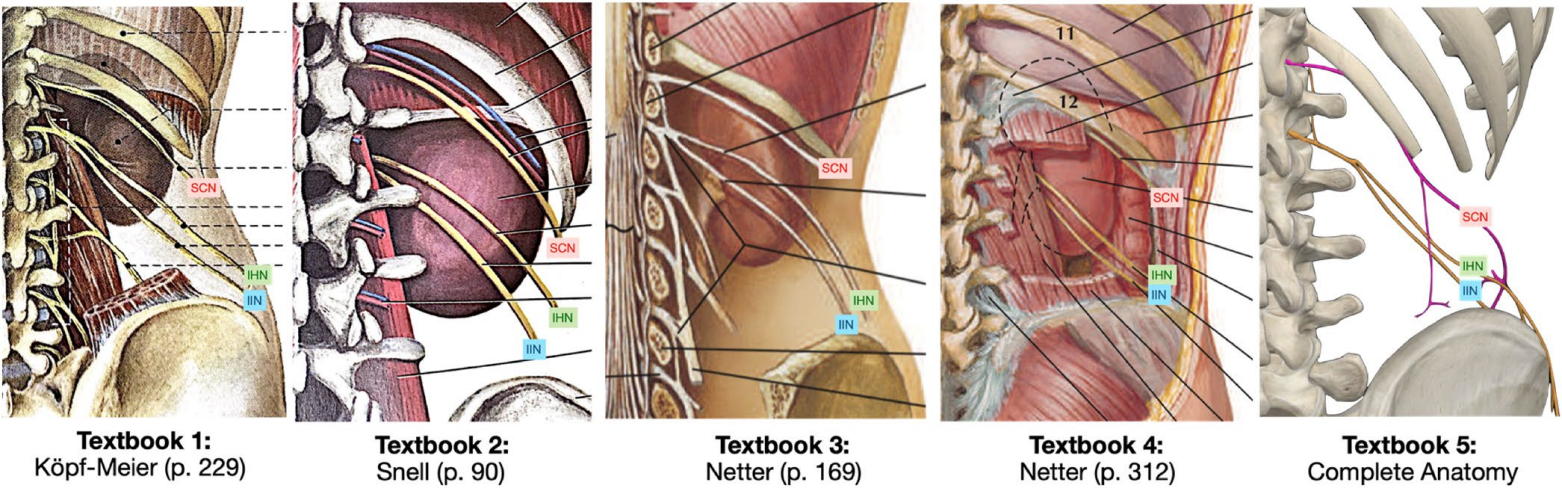
Five textbook images (Köpf‐Maier 2005; Snell 1978; Netter 2019; 3D4Medical 2019).

We aimed to determine the mean locations of the SCN, IHN, and IIN using textbook images that exposed the area between the 12th rib and iliac crest from the posterior view (Figure 5) (Köpf‐Maier 2005; Snell 1978; Netter 2019; 3D4Medical 2019).

**FIGURE 6:**
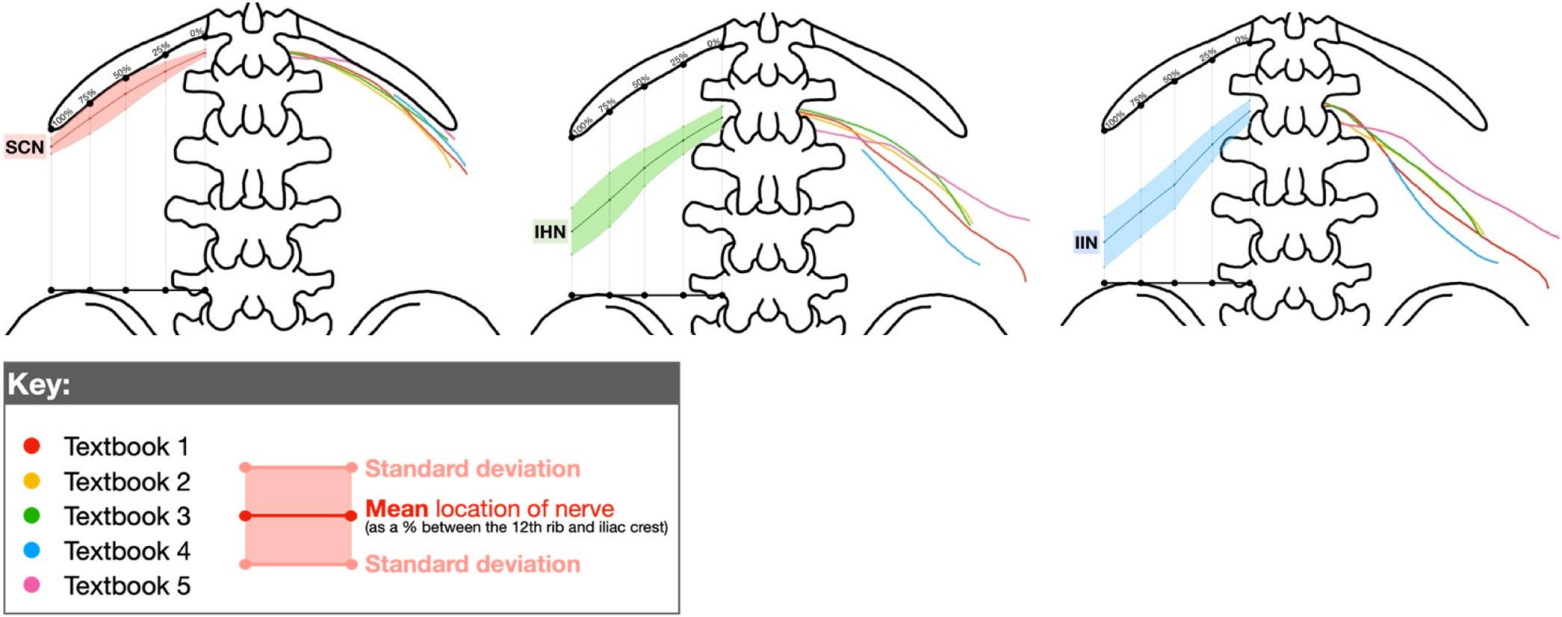
Visual representation of Table 3. The right side of the skeleton traces the course of the SCN, IHN and IIN, with each textbook represented by a different color (key provided). The left side displays the mean measurements and standard deviations.

**FIGURE 7:**
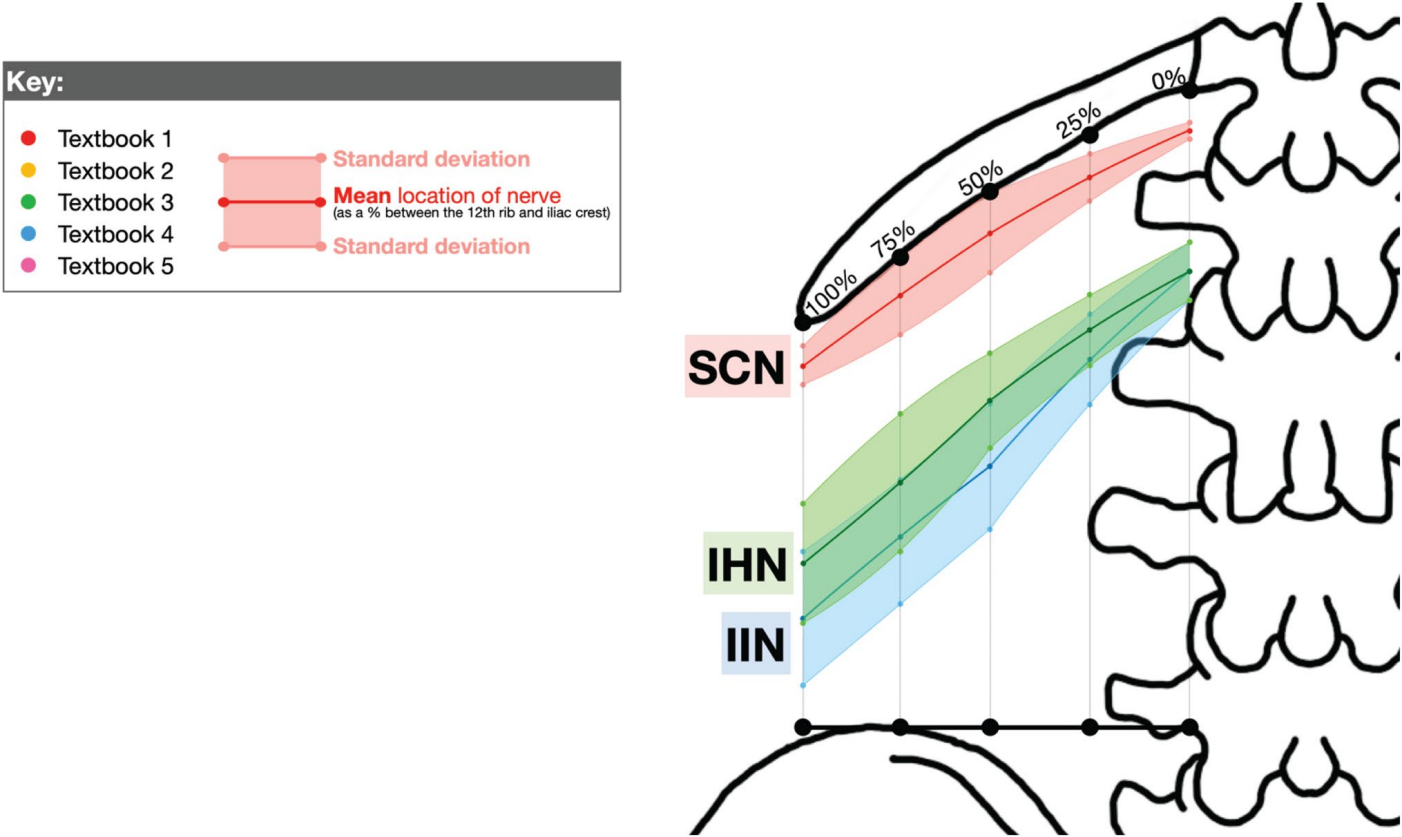
Visual representation of Table 4. The solid red (SCN), green (IHN), and blue (IIN) lines represent the vertical location of the nerves, while the shaded area represents the standard deviations.

Table 3 presents the vertical measurements (as a percentage between the 12th rib and iliac crest) that were recorded at 0%, 25%, 50%, 75%, and 100% along the 12th rib. Hidden nerves (black boxes) and outliers, values ≥ 1.5 times outside the interquartile range (red numbers) were excluded from the analysis. The right side of Figure 6 traces the course of the SCN, IHN, and IIN, with each textbook represented by a different color (key provided). The left side displays the mean measurements and SDs. Table 4 represents the mean and SDs for the locations of the SCN, IHN, and IIN, and are visually represented in Figure 7.

**TABLE 3:** Raw data tables.

SCN	Horizontal measurement (% along the 12th rib)				
Textbook	0	25	50	75	100
1	5	8	6	7	17
2	6	14	18	23	12
3	7	6	6	8	
4				6	10
5	8	3	0	0	4
**Mean**	**7**	**8**	**4**	**7**	**11**
SD	1	5	3	1	5

IHN	Horizontal measurement (% along the 12th rib)				
Textbook	0	25	50	75	100
1	27	29	38	43	56
2		41	49	60	60
3	25	27	28	33	54
4			48	68	83
5	34	35	34	37	42
**Mean**	**29**	**33**	**39**	**48**	**59**
SD	5	6	9	15	15

IIN	Horizontal measurement (% along the 12th rib)				
Textbook	0	25	50	75	100
1	27	34	50	60	71
2		50	66		
3	25	34	42	51	79
4			61	79	93
5	34	35	37	46	52
**Mean**	**29**	**34**	**51**	**59**	**74**
SD	5	1	12	15	17

*Note*: Measuring the location of the SCN, IHN, and IIN as represented by five textbooks. Location is measured as vertical percentages between the 12th rib and iliac crest at 0%, 25%, 50%, 75%, and 100% along the 12th rib. Red numbers represent outliers, and black boxes represent hidden nerves.

**TABLE 4:** Processed data table.

Mean (±SD)	Horizontal measurement (% along the 12th rib)				
Nerve	0	25	50	75	100
SCN	**7** ± 1	**8** ± 5	**4** ± 3	**7** ± 1	**11** ± 5
IHN	**29** ± 5	**33** ± 6	**39** ± 9	**48** ± 15	**59** ± 15
IIN	**29** ± 5	**34** ± 1	**51** ± 12	**59** ± 15	**74** ± 17

*Note*: Representing the mean location (±standard deviation) of the SCN, IHN, and IIN as represented across five textbooks.

### 3.2 Cadaveric Nerve Mapping

**FIGURE 8:**
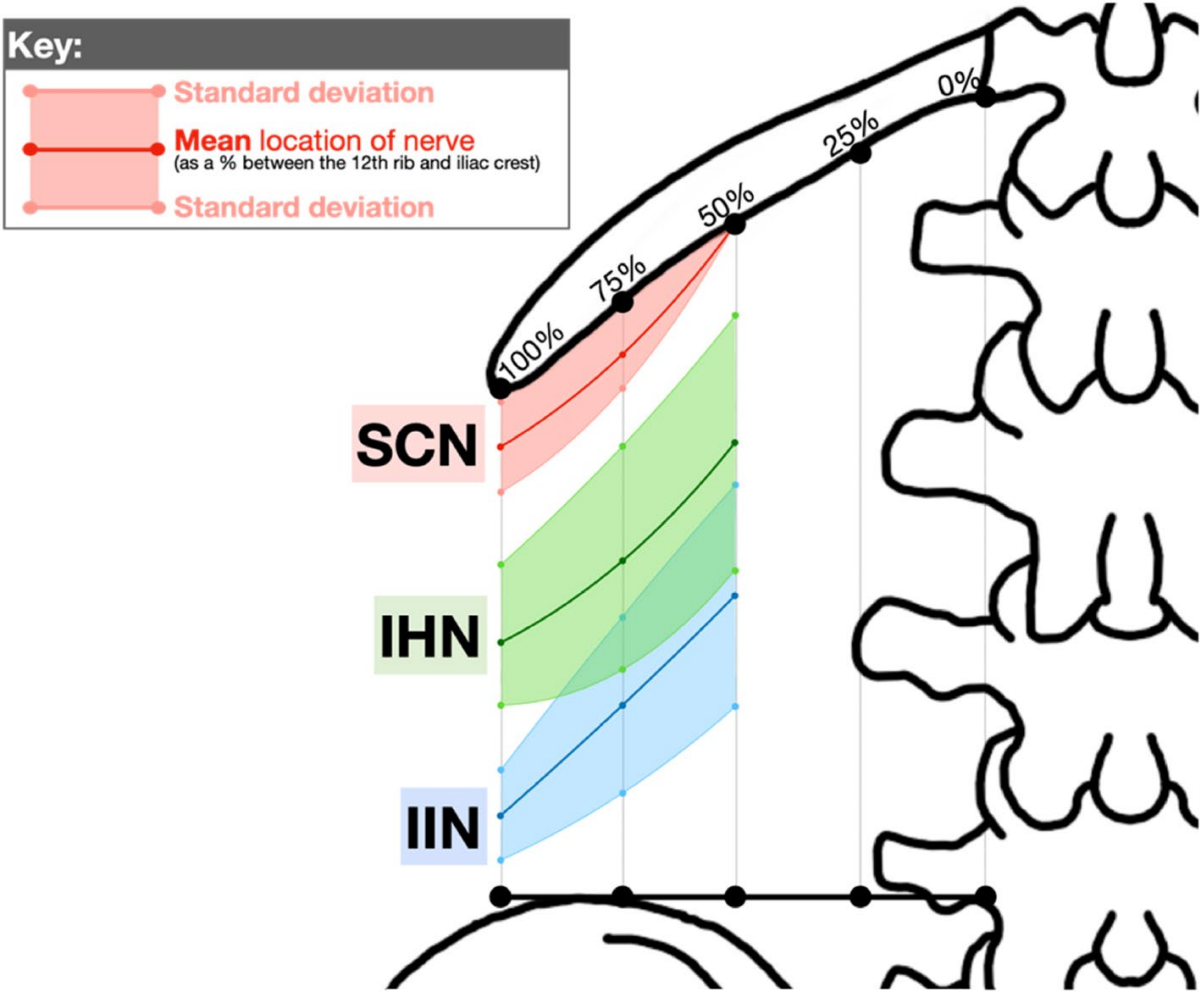
All three nerves. Visual representation of Table 6. The same description as Figure 7.

We compared nerve courses in 13 cadaveric hips to textbook mapping, recording vertical measurements as percentages between the 12th rib and iliac crest (Table 5). Hidden nerves (black boxes) and outliers (red numbers) were excluded from the analysis. Data for the IIN was not collected for Cadavers 1L, 1R, 2R, and 4R due to protocol optimization and dissection challenges. Figure 8 displays these measurements graphically (Table 6).

**TABLE 5:** Raw data tables.

SCN	Horizontal measurement (% along the 12th rib)		
Cadaver ID and hip side	50	75	100
1L		7	0
1R	0	0	0
2R	0	8	18
3L	0	0	0
3R	0	0	11
4L	0	7	18
4R	0	0	12
5L	8	10	24
5R	0	6	11
6L	0	23	18
6R	5	12	5
7L	0	4	16
7R	0	19	25
**Mean**	**0**	**7**	**12**
SD	0	7	9

IHN	Horizontal measurement (% along the 12th rib)		
Cadaver ID and hip side	50	75	100
1L		47	34
1R	6	24	45
2R	39	52	53
3L	13	15	25
3R	13	20	25
4L	20	27	50
4R	18	29	41
5L	56	56	53
5R	65	78	79
6L	55	59	56
6R	39	58	58
7L	25	40	65
7R	31	49	66
**Mean**	**32**	**43**	**50**
SD	19	19	16

IIN	Horizontal measurement (% along the 12th rib)		
Cadaver ID and hip side	50	75	100
1L			
1R			
2R			
3L	49	56	72
3R	25	40	69
4L	40	65	83
4R			
5L	65	71	81
5R	85	91	90
6L	69	80	92
6R	60	78	95
7L	48	56	100
7R	52	74	82
**Mean**	**55**	**68**	**85**
SD	17	15	10

*Note*: Measuring the location of the SCN, IHN, and IIN as represented by 13 cadaveric hips. Location is measured as vertical percentages between the 12th rib and iliac crest at 50%, 75%, and 100% along the 12th rib. Red numbers represent outliers, and black boxes represent hidden nerves.

**TABLE 6:** Processed data table.

Mean (±SD)	Horizontal measurement (% along the 12th rib)		
Nerve	50	75	100
SCN	**0** ± 0	**7** ± 7	**12** ± 9
IHN	**32** ± 19	**43** ± 19	**50** ± 16
IIN	**55** ± 17	**68** ± 15	**85** ± 10

*Note*: Representing the mean location (± standard deviation) of the SCN, IHN, and IIN as represented across five textbooks.

**FIGURE 9:**
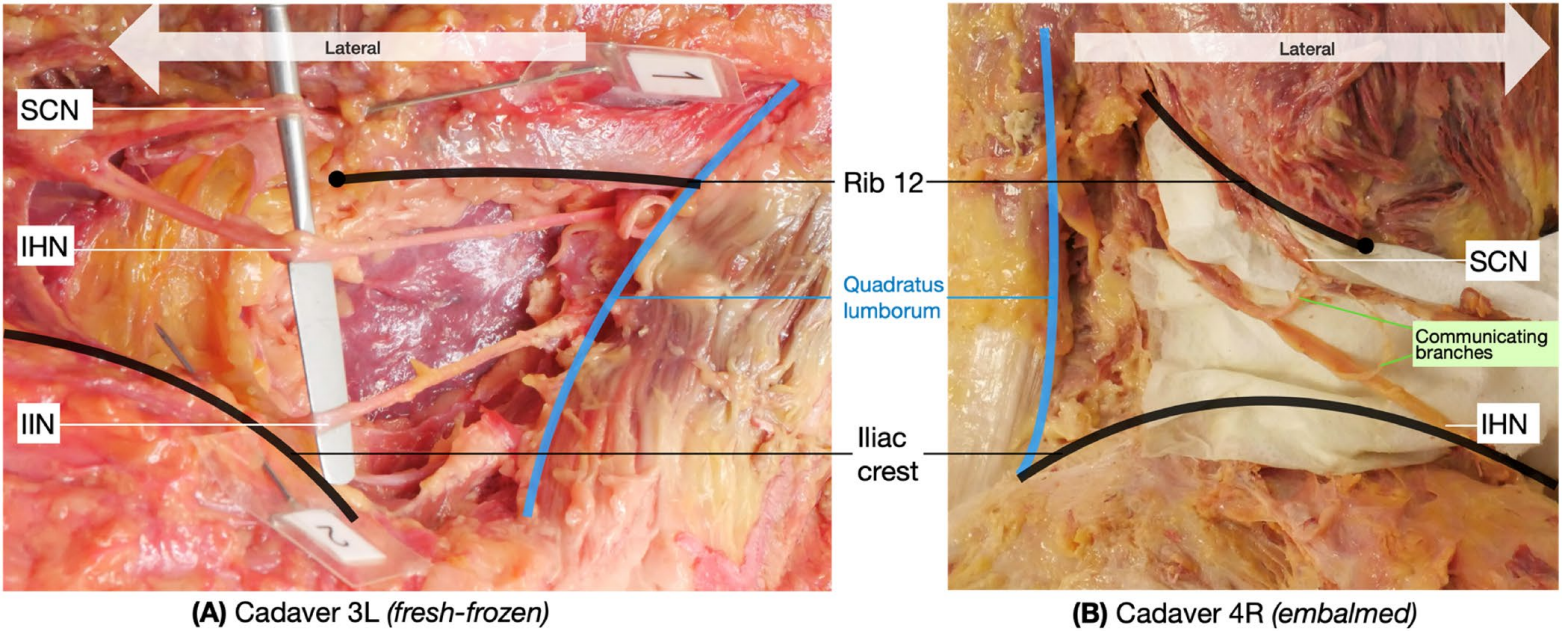
Example of dissections of cadaver 3L and cadaver 4R.

Table 7 outlines qualitative observations of branching patterns, with variability beyond the 12th rib observed in 10 of 13 cadavers. Figure 9 shows labeled examples of two dissections. See the Supporting Information for additional photographs.

**TABLE 7:** Qualitative descriptions of cadaveric dissections (*n* = 13).

Cadaver ID and hip side	Which nerves were identifiable?	Qualitative observation	At what percentage along the 12th rib does this occur?
1L	SCN and IHN only	**SCN and IHN** cross beyond 100% and continue as separate nerves	Beyond 100%
1R			
2R		Single branch of **SCN** splits into two branches	Beyond 100%
3L	SCN, IHN, and IIN	**SCN and IHN** coalesce to form one branch	Beyond 100%
3R			
4L		Single branch of **SCN** splits into multiple branches	Beyond 100%
4R		Two communicating branches between **SCN and IHN**	At 75% and 100%
5L		Two branches of **SCN** coalesce to form one branch	Beyond 100%
5R			
6L		Two branches of **SCN** visible and continue to remain separate	From 50% onward
6R		Two branches of **SCN** visible and continue to remain separate	From 50% onward
7L		**SCN, IHN and IIN** all coalesce and form multiple branches	Beyond 100%
7R		**IHN and IIN** cross and continue as separate nerves	Beyond 100%

Table 8 compares textbook and cadaveric measurements. Unpaired *t*‐tests were used, except for the SCN at 50%, where a *Mann–Whitney U* test was used due to nonnormal distribution. These statistical tests showed no significant difference (*p* > 0.05), confirming the accuracy of textbook nerve mapping.

**TABLE 8:** Textbook and cadaveric mean vertical measurements of SCN, IHN, and IIN nerves as a percentage between the 12th rib and iliac crest (±standard deviations) at 50%, 75%, and 100% along the 12th rib.

Nerve		SCN			IHN			IIN		
Horizontal measurements as a % along 12th rib		50%	75%	100%	50%	75%	100%	50%	75%	100%
Vertical measurements as a % between 12th rib and iliac crest (±SD)	Textbooks (*n* = 5)	**4** ± 3	**7** ± 1	**11** ± 5	**39** ± 9	**48** ± 15	**59** ± 15	**51** ± 12	**59** ± 15	**74** ± 17
	Cadavers (*n* = 13)	**0** ± 0	**7** ± 7	**12** ± 9	**32** ± 19	**43** ± 19	**50** ± 16	**55** ± 17	**68** ± 15	**85** ± 10
*p*		0.112	0.931	0.768	0.798	0.559	0.293	0.694	0.351	0.168
Mean position of the nerves (textbooks and cadavers combined)		**2**	**7**	**12**	**36**	**46**	**55**	**53**	**64**	**80**

### 3.3 Defining the Ideal Injection Site

**FIGURE 10:**
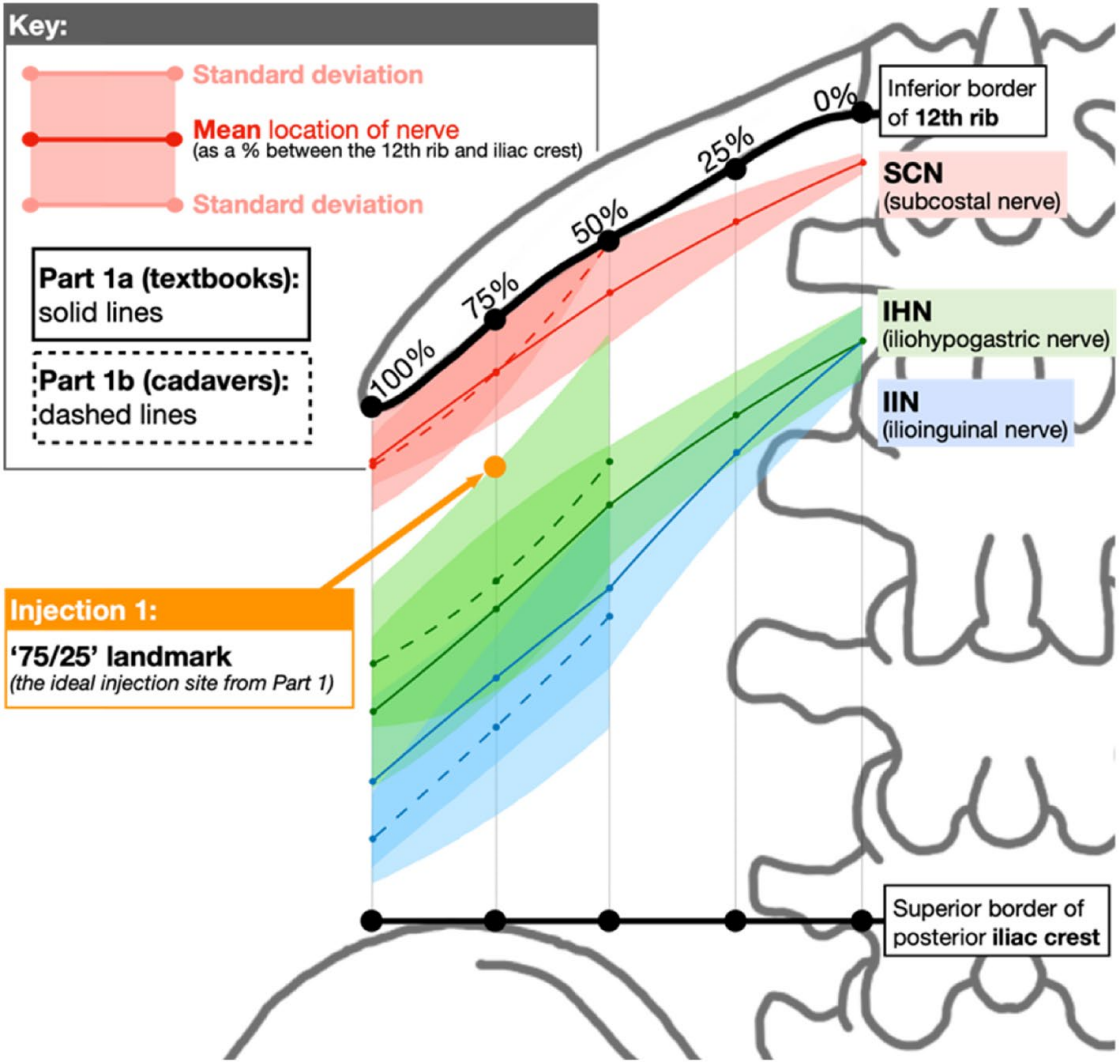
Direct comparison of textbook (solid lines) and cadaveric (dashed lines) measurements.

Based on textbook and cadaveric mapping, the ideal injection site for PNBs to target the SCN, IHN, and IIN is at the “75/25” landmark: 75% horizontally along the 12th rib between the T12 costovertebral joint and the tip of the 12th rib and 25% vertically down between the 12th rib and iliac crest. This is labeled as “Injection 1” in Figure 10, which overlays the textbook (Figure 7) and cadaver (Figure 8) measurements.

The 75% point was identified as the ideal horizontal landmark for three reasons. Firstly, a landmark more lateral than 50% was preferred due to the narrowing space between the inferiorly curving 12th rib with the superiorly curving iliac crest (Manzaneda et al. 2023), increasing the chance of targeting all three nerves with one injection. Secondly, a landmark more medial to 100% was preferred to avoid the area of high anatomical variability in branching patterns observed beyond the 12th rib in Table 6 and supported by Drakonaki et al. (Drakonaki et al. 2022) Third, 75% was easily identifiable on surface anatomy, lying midway between the palpable quadratus lumborum (50%) and the tip of the 12th rib (100%).

**FIGURE 11:**
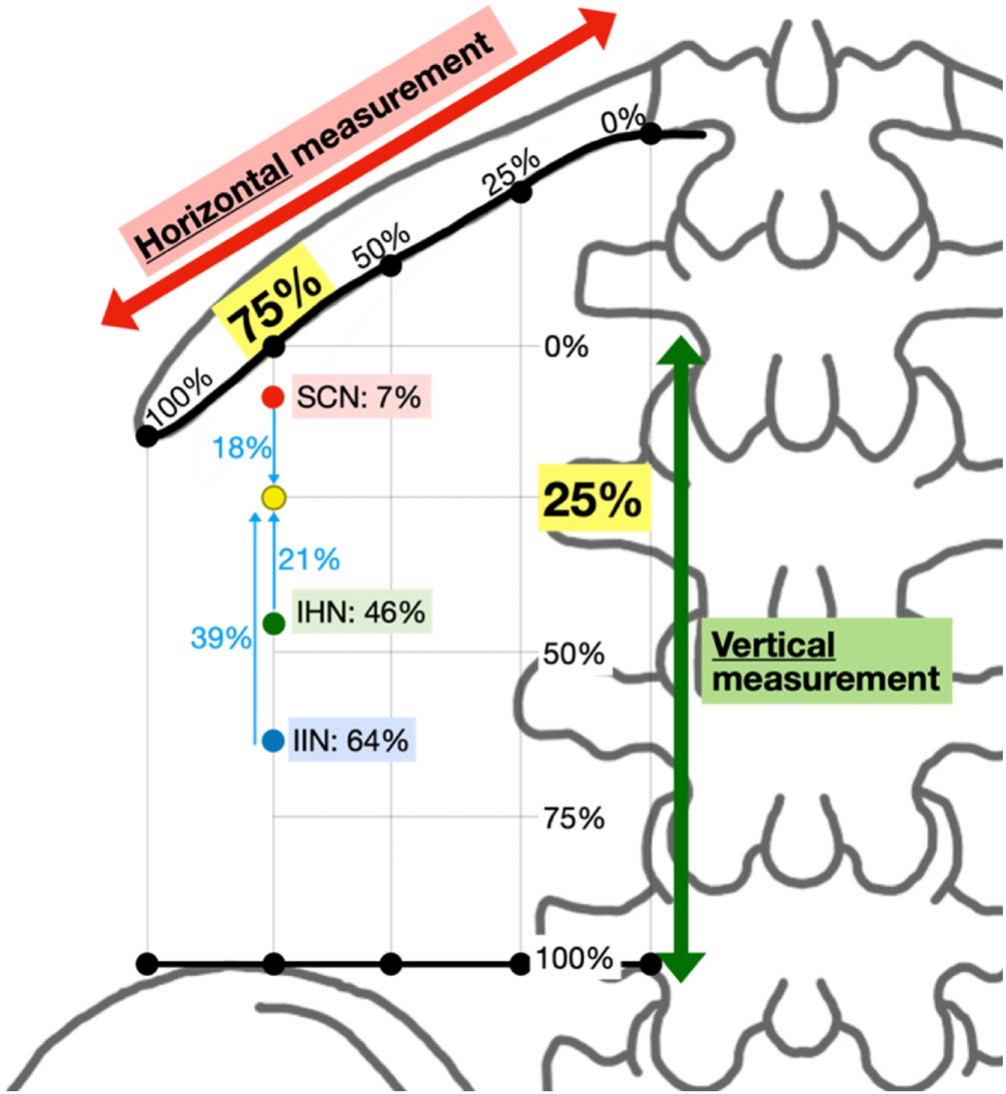
Defining 25% as the ideal vertical landmark.

The 25% point was identified as the ideal vertical landmark. Using multiples of 25, this was predicted to be the easiest percentage to estimate using surface anatomy between the 12th rib and iliac crest that would target all three nerves. Referring to the mean values from Table 8, 25% was 18% inferior to SCN, 21% superior to IHN and 39% superior to IIN (Figure 11). An injection at 50% may have been too inferior to target the SCN while 0% may have been too superior to target the IIN.

### 3.4 Testing the “75/25” Injection Site

Injection 1 stained the SCN in 6/6, IHN in 4/6 and IIN in 2/6 hips (Table 9). The IHN was not stained in cadavers 5L and 5R due to a technique error where the injection was too close to the 12th rib, and not exactly 25% vertically down. Therefore, except for this error, the “75/25” landmark consistently targeted the SCN and IHN but not the IIN.

**TABLE 9:** Staining of the SCN, IHN, and IIN by injection 1 at the “75/25” landmark on cadavers 5–7.

Cadaver ID and hip side	Have the nerves been stained by injection 1 at the “75/25” landmark?		
	SCN	IHN	IIN
5L	Yes	No	No
5R	Yes	No	No
6L	Yes	Yes	No
6R	Yes	Yes	No
7L	Yes	Yes	Yes
7R	Yes	Yes	Yes
Total (number out of 6 hips)	**6**	**4**	**2**

### 3.5 Exploring the Nerves in the Posterolateral Hip Area

**FIGURE 12:**
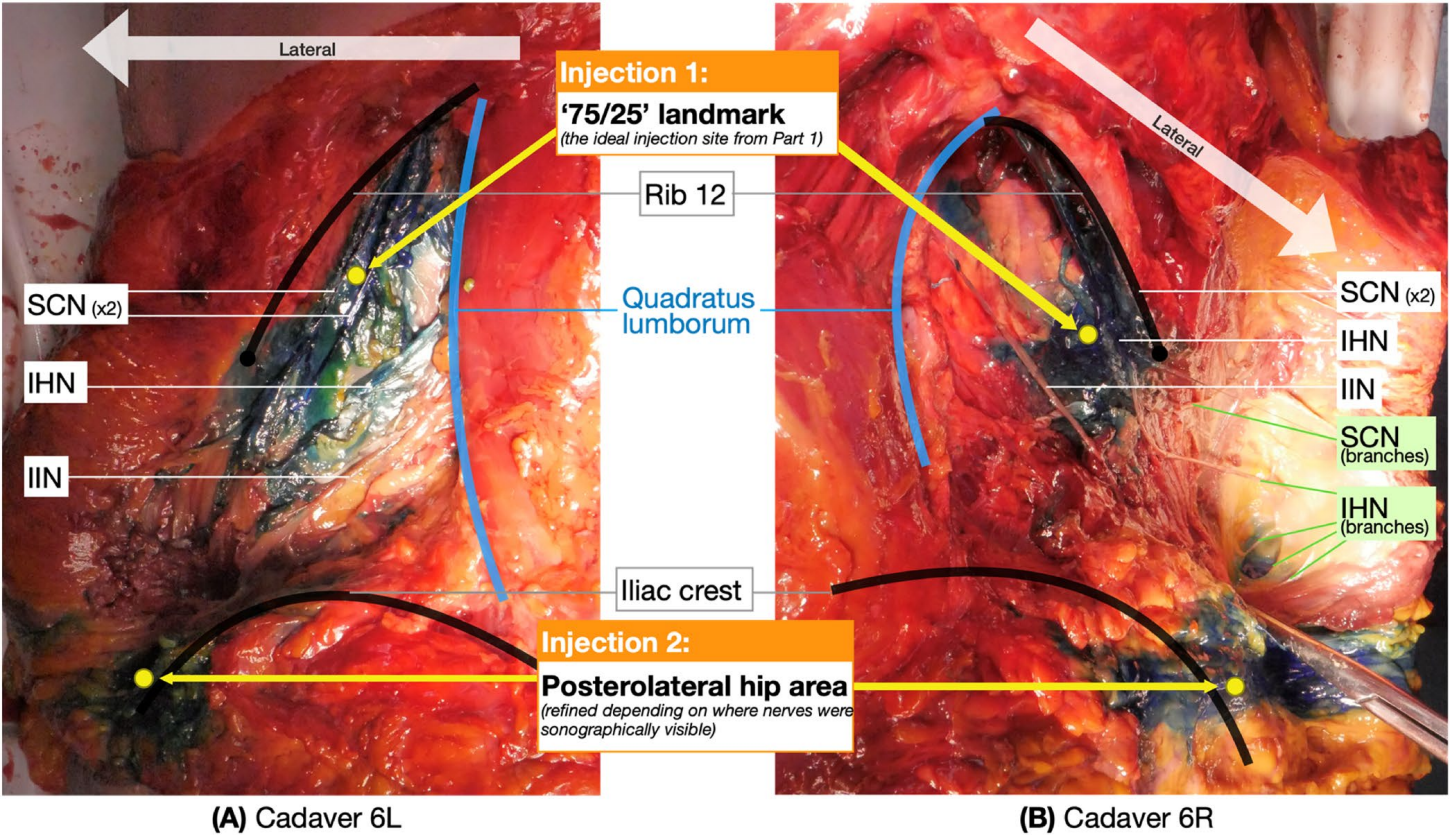
Example of dissections of cadaver 6L and 6R after Injections 1 and 2.

The secondary aim was to identify the cutaneous nerves in the posterolateral hip area that branch over the iliac crest posterior to the ASIS, as represented in Figures 1, 2, 3 (Dalley and Agur 2024; Abrahams et al. 2019) and labeled as “Injection 2” in Figure 12. This landmark served as the initial placement point for the ultrasound probe; however, this was refined depending on where cutaneous nerves were sonographically visible.

Injection 2 stained the branches of the SCN in 1/6, the IHN in 4/6, and IIN in 4/6 hips (Table 10). In Cadavers 5R and 7R, the injection failed to stain any of the three nerves due to a technique error, injecting too inferiorly to the iliac crest, thus not accurately targeting the nerves. Therefore, except for this error, sonographically identifiable cutaneous nerves in the posterolateral hip area were branches of the IHN and IIN and not the SCN.

**TABLE 10:** Staining of SCN, IHN, and IIN branches by injection 2 in the posterolateral hip area on cadavers 5–7.

Cadaver ID and hip side	Do the nerves branch to injection 2 in the posterolateral hip area? (at the level of iliac crest posterior to ASIS)		
	SCN	IHN	IIN
5L	No	Yes	Yes
5R	No	No	No
6L	No	Yes	Yes
6R	No	Yes	Yes
7L	Yes	Yes	Yes
7R	No	No	No
Total (number out of 6 hips)	**1**	**4**	**4**

## 4 Discussion

We showed textbook mapping to accurately reflect the cadaveric mapping of the SCN, IHN, and IIN. Cadavers showed high variability in branching patterns, especially beyond the tip of the 12th rib. “75/25” was identified as the ideal injection site for targeting these three nerves. We confirmed the effectiveness of the “75/25” landmark in targeting the SCN (6/6) and IHN (4/6) but not the IIN (2/6). However, it was the IHN (4/6) and IIN (4/6) that branched to the posterolateral hip area, not the SCN (1/6).

Textbook mapping accurately reflected cadaveric findings for the SCN, IHN, and IIN, showing the SCN originating near the 12th rib (7% ± 1%) and the IHN and IIN originating more inferiorly (29% ± 5%) (Table 4). These findings were consistent with literature describing the SCN originating from T12 and the IHN and IIN originating from L1 (Table 1) (Standring, Anand, and Tunstall 2021; Abrahams et al. 2019). The courses of the IHN and IIN displayed greater variability with overlapping SDs compared to the SCN (Figure 7). Despite differing origins and courses between the SCN and IHN, communicating branches and coalescing/crossing patterns occurred between the SCN and IHN in 4 out of 13 hips (Table 7), moderately supported by similar literature findings (Vadhanan, Hussain, and Prakash 2019; Alonso et al. 2017). Other variations reported in the literature include individuals lacking an IHN or IIN (Standring, Anand, and Tunstall 2021; Anloague and Huijbregts 2009) or coalescing patterns between the IHN and IIN occurring near the iliac crest (Apaydin and Bozkurt 2015).

Textbooks often simplify nerve mapping for educational clarity, which may not fully represent the true anatomical variability (Netter 2019). This study found textbooks to map the SCN, IHN, and IIN with straighter courses (Figure 9) compared to varied branching observed in cadavers (Table 7). Textbooks also represent nerves with more space between landmarks for easier identification (Tubbs, Shoja, and Loukas 2016). For example, Textbook 2 shows the nerves with more spacing and further from the 12th rib, thus resulting in three outliers (displayed in red text in Table 3).

**FIGURE 13:**
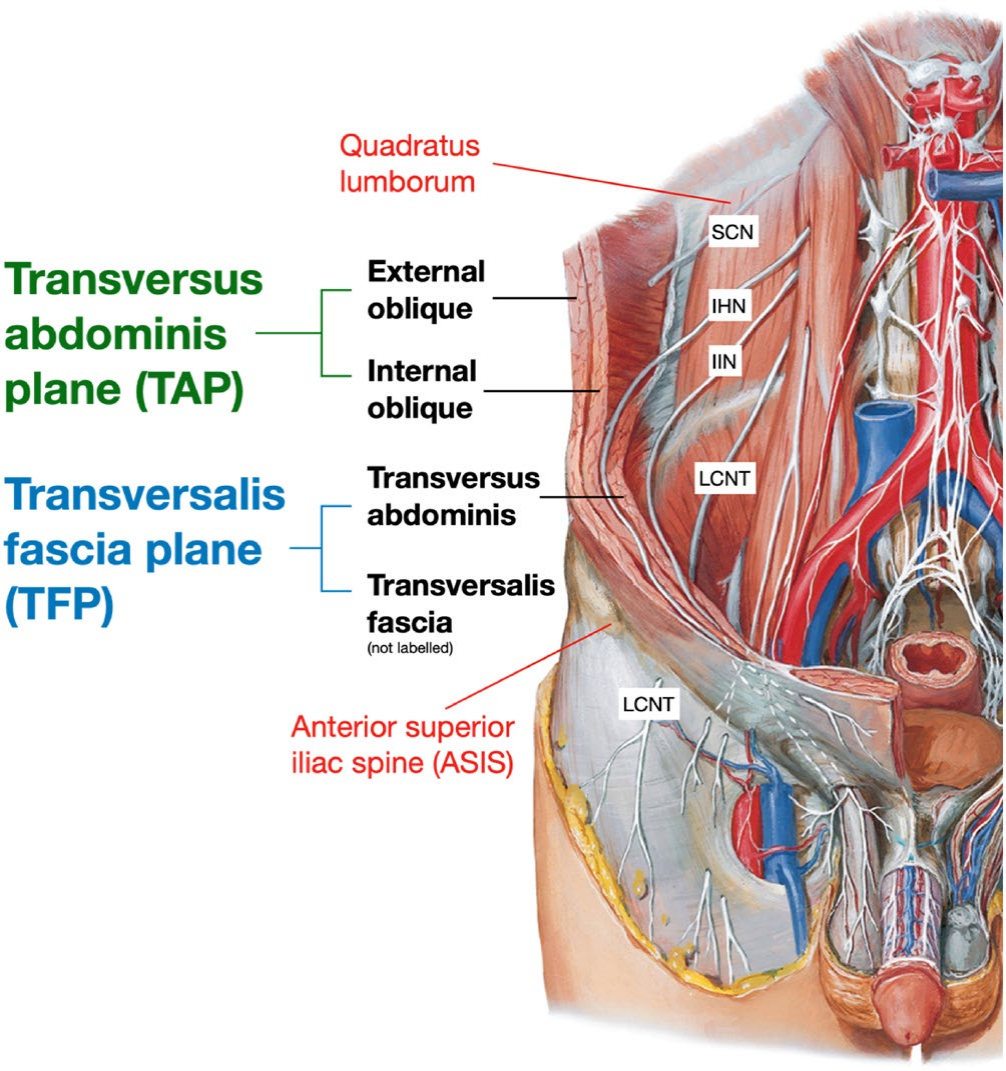
Different planes which nerves may be located in. Adapted from Netter's Atlas of Human Anatomy p. 391 (Netter 2019).

We showed that ultrasound‐guided dye injections at the “75/25” landmark consistently stained the SCN (4/6) and IHN (4/6) but not the IIN (2/6). The IIN may not have been consistently stained for three reasons. First, the 25% vertical landmark may have been too close to the 12th rib, missing the IIN which is 39% inferior (Figure 11). Second, the 5 mL dose of 0.25% methylene blue might have been insufficient. However, a larger dose poses clinical risks such as bleeding, infection, nerve injury, or local anesthetic systemic toxicity (Schwenk and Mariano 2018). Third, the IIN may exist in a different plane than the SCN and IHN. The literature has identified the IIN to originate in the transversalis fascia plane and then laterally enter the transversus abdominis plane where the SCN and IHN run (Figure 13) (Netter 2019); however, this precise point when the IIN changes planes, varies in the literature (Chin et al. 2012; Apaydin and Bozkurt 2015; Drakonaki et al. 2022). On the contrary, the IIN was successfully stained in Cadavers 7L and 7R, potentially due to the donor's smaller vertical distance between the 12th rib and iliac crest, suggesting that smaller distances might increase the likelihood of staining all three nerves (Manzaneda et al. 2023).

The ultrasound‐guided injection at the iliac crest posterior to the ASIS showed that nerves in this area were branches of the IHN (4/6) and IIN (4/6) but not the SCN (1/6). This confirms the IHN's role in innervating the posterolateral hip area, supported by strong evidence (Glenister and Sharma 2022; Standring, Anand, and Tunstall 2021; Dalley and Agur 2024; Abrahams et al. 2019), and suggests moderate involvement of the IIN (Chin et al. 2012; Apaydin and Bozkurt 2015; Drakonaki et al. 2022). The SCN's role in this area was inconsistent with previous literature (Figure 3) (Dalley and Agur 2024; Maigne, Maigne, and Guerin‐Surville 1986; Nielsen et al. 2019), though it was present in Cadaver 7L (Table 10) due to coalescence with the IHN and IIN (Table 7). This coalescing pattern, while not extensively described, has been noted in 20% of cases in the literature (Manolakos et al. 2022).

**FIGURE 14:**
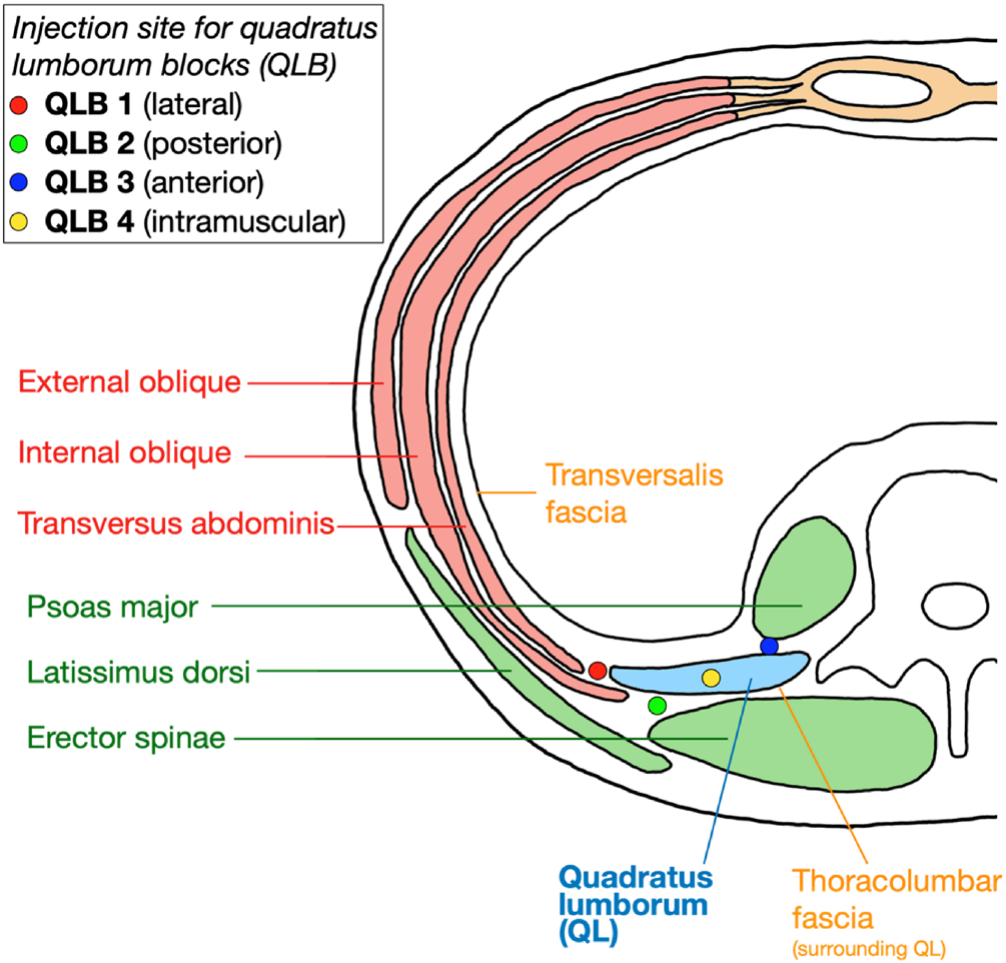
Cross‐section of abdomen, identifying the four sites for needle insertion for QLB. Adapted by Akerman et al. (Akerman, Pejčić, and Veličković 2018).

This study highlights that a PNB targeting the IHN and IIN is more effective for posterolateral hip anesthesia, compared to including the SCN at the “75/25” landmark, which inconsistently blocks the IIN. PNBs that target the IHN and IIN are already used in abdominal surgery. For example, the quadratus lumborum block (QLB) is performed near the 12th rib at any of the four needle insertion sites (Figure 14) (Akerman, Pejčić, and Veličković 2018) and provides analgesia in T7‐L1 dermatomes (Dhanjal and Tonder 2019). Its effectiveness in hip replacement surgery varies in the literature, with some studies showing reduced pain and opioid use (Huda and Minhas 2022), while others show no changes (Brixel et al. 2021). Furthermore, a 2016 case study noted L2 dermatomal sensory loss and hip flexion weakness for 18 h postsurgery following a lateral QLB (Wikner 2017).

**FIGURE 15:**
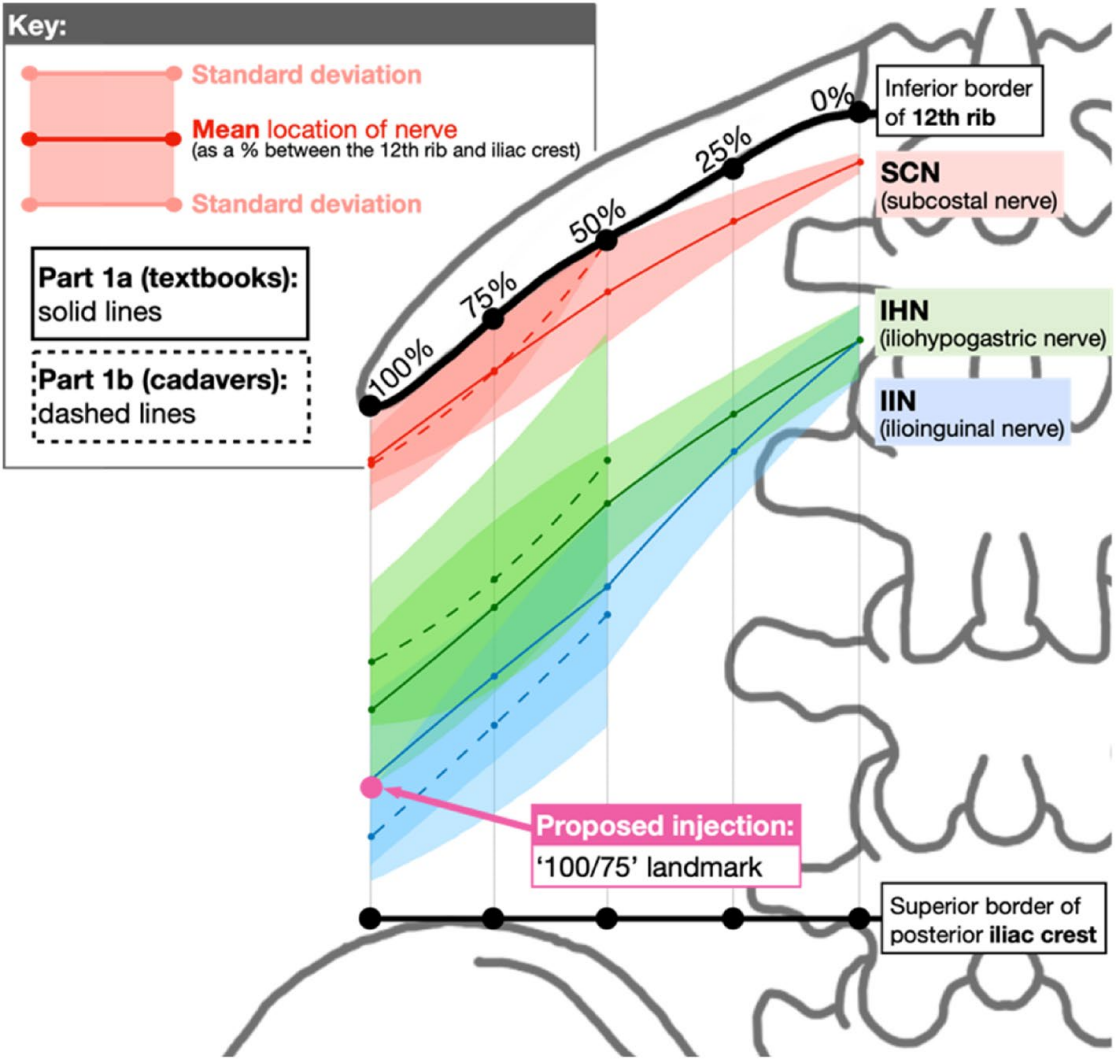
New injection site proposed at the “100/75” landmark to target the IHN and IIN.

To address the limitations of a QLB, a PNB at the “100/75” landmark is proposed for hip replacement surgery (Figure 15). This landmark targets the distal branches of the IHN and IIN, rather than their L1 nerve root, therefore minimizing sensory and motor loss (O'Flaherty, McCartney, and Ng 2018). This landmark also effectively blocks these nerves before their variable branching patterns (Table 7) (Drakonaki et al. 2022). This ultrasound‐guided approach may use the “100/75” landmark as an initial placement point for the ultrasound probe, refining the site of needle insertion depending on where nerves are sonographically visible.

### 4.1 Strengths and Limitations

This study has several strengths. Using percentages instead of metric units for nerve location allowed for easier comparison across cadavers by accounting for anatomical variations, such as rib length and spacing (Manzaneda et al. 2023). The similarity between cadaveric and textbook measurements confirmed accurate dissection techniques. Additionally, the novel finding that the IIN, rather than the SCN, innervates the posterolateral hip was another key strength.

However, there were also limitations. Inconsistent photographic angles during cadaveric dissections affected measurement accuracy. Distinguishing nerves from the surrounding tissues proved challenging, with no reliable method to confirm nerve identity. Nerves lost their natural position after dissection, further affecting measurements. The small sample size of five textbook images and 13 cadaveric hips limited the findings. Finally, the cadaveric study design may not be generally valid, as dye spread in cadavers differs from local anesthetic in living patients (Anagnostakou et al. 2022) and factors such as the advanced age of donors (88 ± 8.5 years) and anatomical variations such as abnormal rib counts (Guttentag and Salwen 1999), must also be considered.

## 5 Conclusions

This study highlights that textbook mapping of the SCN, IHN, and IIN is not universally accurate. The key nerves to target for postoperative analgesia after hip replacement surgery via the posterior or lateral approach are the IHN and IIN as these are the nerves that branch over the iliac crest posterior to the ASIS, innervating the posterolateral hip area. While the “75/25” landmark effectively blocks the SCN and IHN, it fails to consistently block the IIN. A more distal PNB at the “100/75” landmark is recommended to reliably target both the IHN and IIN. In clinical practice, combining a “100/75” block with an LCNT block may optimize pain management and recovery.

Future research should focus on translating these cadaveric findings to clinical practice by assessing the effectiveness of the novel “100/75” block in patients after hip replacement surgery. A randomized controlled trial could compare two groups: one receiving the current LCNT block alone and the other receiving an LCNT with an additional “100/75” block. The primary outcome would be pain scores, and the secondary outcome would be length of hospital stay.

## Ethics Statement

Ethical approval was granted by the University of Nottingham Ethics Committee. Research adhered to the UK Human Tissue Act (2004), with all donors providing consent for use and imaging. The University of Nottingham Anatomy Suite is licensed by the Human Tissue Authority (No. 12085).

## Conflicts of Interest

The authors declare no conflicts of interest.

## Supporting information


**Data S1.** Supporting Information.
