# Supplementary material for: Optimizing Peripheral Nerve Block Placement in Hip Surgery: A Cadaveric Study Mapping the Posterior Cutaneous Innervation
Source: Clin Anat. 2025 Feb 14;38(8):836–51. doi: 10.1002/ca.24262 (PMC12522074; doi:10.1002/ca.24262)

## Supplementary Materials:

## Dissection photos for Cadavers 1-7

Below are the labelled dissection photos for the left (L) and right (R) hip of cadavers in Part 1b and 2. All cadavers were fresh-frozen except Cadaver 4L which was embalmed. Labels depict the subcostal nerve (SCN), iliohypogastric nerve (IHN), ilioinguinal nerve (IIN), the lateral border of the quadratus lumborum (QL), the inferior border of the 12^th^ rib and the superior border of the posterior iliac crest. Dissection photos for cadavers 3L, 4R, 6L and 6R have been excluded as they are featured in Figures 8 and 11 of the paper.


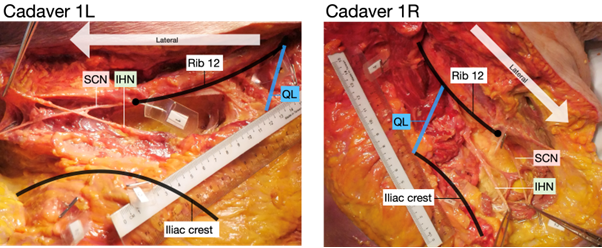


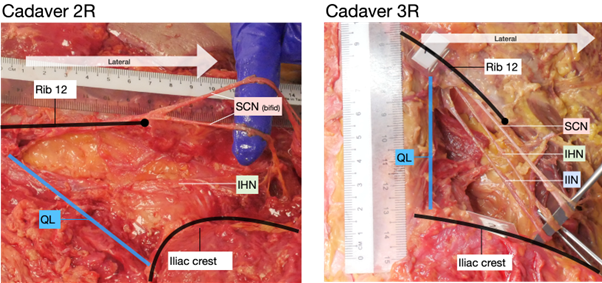


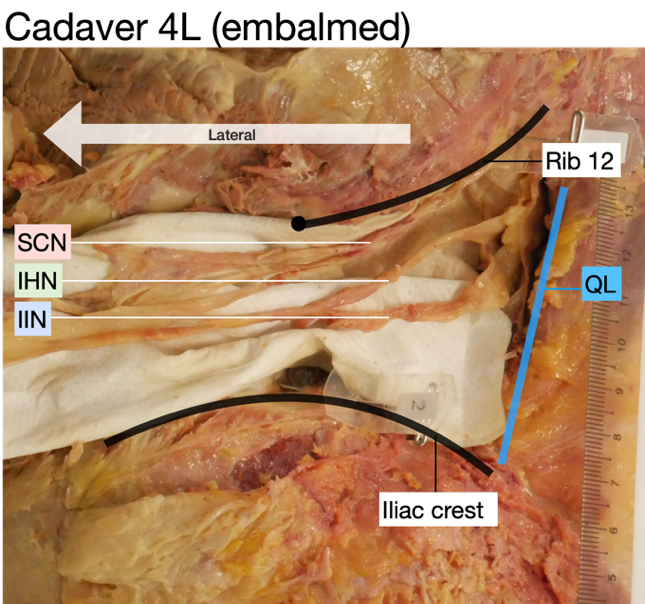

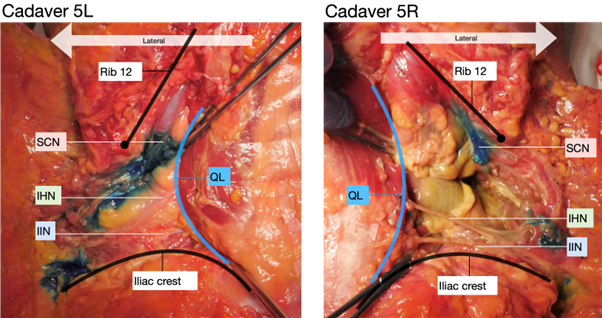

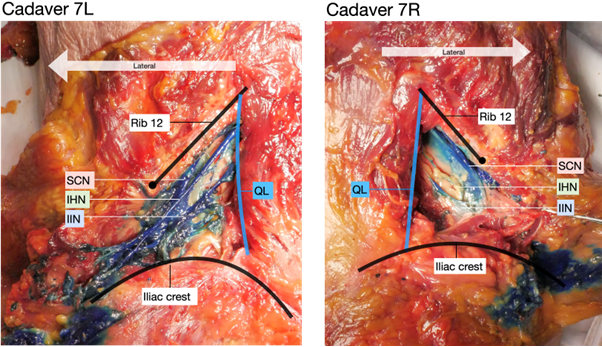

Supplement: Supplementary file 1 — Data S1. Supporting Information. [file CA-38-836-s001.docx]
